# Draft genome data of *Fusarium proliferatum* TQN5T isolated from *Dysosma versipellis*

**DOI:** 10.1016/j.dib.2026.113040

**Published:** 2026-06-27

**Authors:** Nhung Thi Doan, Ha Thi Hong Nguyen, Quang Ho Tran, Tung Ngoc Quach, Ngoc Bich Pham

**Affiliations:** a Institute of Biology (IB), Vietnam Academy of Science and Technology (VAST), Hanoi, 100000, Vietnam; b Graduate University of Science and Technology (GUST), Vietnam Academy of Science and Technology (VAST), Hanoi, 100000, Vietnam

**Keywords:** Genomic analysis, *Fusarium proliferatum*, Endophytic fungi, *Dysosma versipellis*

## Abstract

*Fusarium proliferatum* TQN5T is an endophytic fungus isolated from *Dysosma versipellis* in Tuyen Quang, Vietnam. The genome assembly comprised 443 contigs totalling 47.7 Mb (46.26% GC content, N50: 509,240 bp) with a completeness score of 96.35%. The draft genome contained 14,299 predicted genes (average length 1516.61 bp), along with 339 tRNAs and 84 rRNAs. Genome annotation revealed 1224 CAZy genes with predominant glycoside hydrolases and 60 secondary metabolite biosynthetic clusters (smBGCs) enriched in T1PKS and terpene types, including bioactive compound clusters such as α-acorenol. This genomic dataset provides essential information to support future mechanistic studies of PTOX biosynthesis and cytotoxic activity in *F. proliferatum* TQN5T.

Specifications Table

**Table:** 

Subject	Biology
Specific subject area	Genomic analysis of endophytic fungi isolated *from Dysosma versipellis* in Vietnam
Type of data	Table, Figure, Raw, Analysed, Processed and Deposited
Data collection	Genomic DNA of *Fusarium proliferatum* TQN5T isolated from the root of *D. versipellis* was quantified using Pippin pulse—SAGE and QUBIT 3.0, sequenced by PacBio SEQUEL system, and assembled with HGAP4. Genome quality was assessed using QUAST and BUSCO. Structural annotation was performed using RepeatMasker, BRAKER2, tRNAscan-SE, and barrnap. Functional annotation of COG, KEGG, and GO categories was conducted using eggNOG-mapper, while carbohydrate-active enzymes and secondary metabolite biosynthetic gene clusters were annotated using dbCAN2 and antiSMASH 8.0, respectively. Taxonomic classification was supported by ITS-based phylogenetic analysis using MAFFT and IQ-TREE 2, and whole-genome Average Nucleotide Identity (ANI) was calculated using FungANI.
Data source location	Institute of Biology, Vietnam Academy of Science and Technology, Hanoi, Vietnam
Data accessibility	Repository name: NCBI GenBank Genome accession GCA_033822135.1 Data identification number: BioProject PRJNA837748, BioSample ID SAMN29447699 Direct URL to data: https://www.ncbi.nlm.nih.gov/datasets/genome/GCA_033822135.1/
Related research article	G.T. Nguyen, H.T.H. Nguyen, H.T. Tran, H.T. Tran, A.N. Ho, Q.H. Tran, N.B. Pham, Enhanced podophyllotoxin production of endophyte *Fusarium proliferatum* TQN5T by host extract and phenylalanine, Appl. Microbiol. Biotechnol. 107 (2023) 5367–5378.

## 1 Value of the Data

• This draft genome of *Fusarium proliferatum* TQN5T provides the first comprehensive genomic resource for this endophytic strain. The high-quality assembly and functional annotation reveal the strain's genetic potential for secondary metabolite biosynthesis, including PTOX production and bioactive compound synthesis.
• These data can be used to elucidate PTOX biosynthetic pathways and cytotoxic mechanisms, identify and validate candidate genes for secondary metabolite production, optimize fermentation strategies for pharmaceutical compound yields, conduct comparative genomic analyses with related fungi, discover and characterize novel bioactive compounds through genome mining.

## 2 Background

*Fusarium proliferatum* is a filamentous fungus belonging to the *Fusarium fujikuroi* species complex, widely distributed as both plant pathogen and endophyte across diverse host plants [1]. Endophytic *Fusarium* species have attracted increasing research interest due to their remarkable capacity to synthesize bioactive secondary metabolites with pharmaceutical potential. *Fusarium proliferatum* TQN5T was isolated from *Dysosma versipellis*, a Vietnamese endemic medicinal plant, and was previously demonstrated to produce podophyllotoxin (PTOX) and exhibit cytotoxic activity against cancer cell lines (LU-1 and HepG2) [6]. Despite its biological significance, the genomic basis underlying the metabolic diversity and ecological adaptations of this endophytic strain remains unexplored. Whole-genome sequencing and comprehensive functional annotation of *F. proliferatum* TQN5T were therefore conducted to provide a foundational genomic resource. This dataset enables comparative genomic studies, genome mining for novel bioactive compounds, and future investigations into the biosynthetic pathways and regulatory mechanisms of secondary metabolite production in this endophytic fungus.

## 3 Data Description

**Fig. 1:**
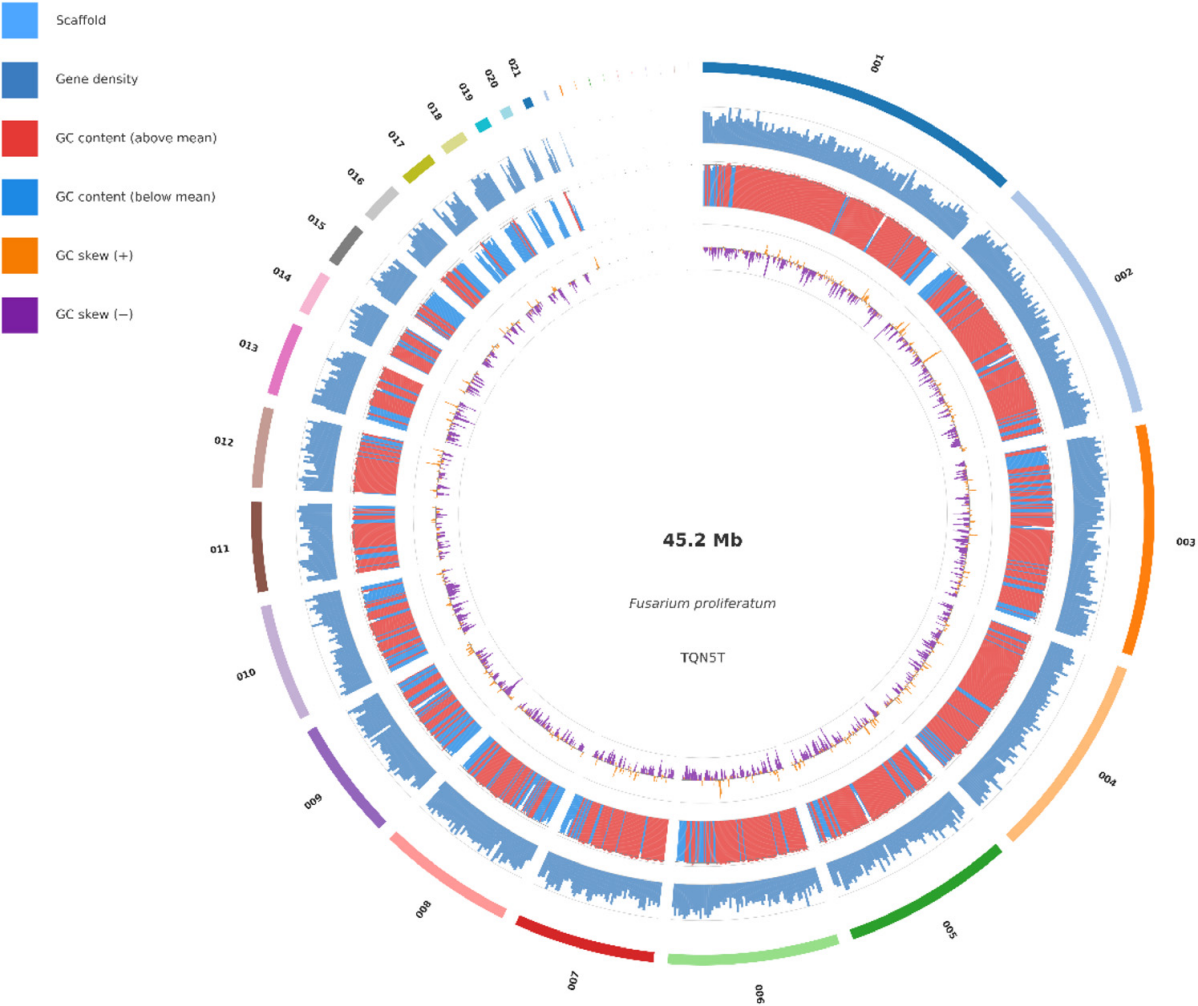
Circular genome map of *Fusarium proliferatum* TQN5T. Tracks from outside to inside represent: scaffolds (colored by scaffold number), gene density, GC content (red: above mean; blue: below mean), and GC skew (orange: positive; purple: negative).

The resulting assembly consisted of 443 sequence contigs with a total length of 47.7 Mb (46.26% GC content) and an N50 length of 509,240 bp, resulting in a completeness score 96.35%. The assembly of *F. proliferatum* TQN5T has been deposited at GenBank under the accession number GCA_033822135.1. A total of 14,299 gene structures were predicted, with an average gene length of 1516.61 bp. Additionally, 339 tRNAs and 84 rRNA were found (Table 1, Fig. 1).

**Table 1:** Summary of genomic features and assembly statistics for strain TQN5T.

Genome assembly features	Values
Total assembly size (Mb)	47.7 Mb
Total assembly size (≥ 1000 bp)	47,733,417
Total assembly size (≥ 50,000 bp)	46,260,612
Number of contigs (≥ 1000 bp)	443
Number of contigs (≥ 50,000 bp)	127
Total number of contigs	443
Largest contig (bp)	1882,326
GC-content (%)	46.26
N50 contig length (bp)	509,240
L50 contig count	32
Total length (bp)	47,733,417
Genome coverage	195x
***Completeness evaluation (%)***	
Completeness	96.35
Complete and single-copy BUSCOs	96
Complete and duplicated BUSCOs	0.4
Fragmented BUSCOs	0.8
Missing BUSCOs	2.6
***Repeat annotation***	
Total repeat length (bp)	545,741
Total repeat content (%)	1.17
Simple repeats (elements / bp / %)	7876 / 498,304 / 1.06
Low complexity (elements / bp / %)	959 / 47,437 / 0.10
***Genomic annotation***	
Genes (total)	14,299
tRNA	339
rRNA (5S, 5.8S, 18S, 28S)	84 (58, 9, 9, 8)

Repeat annotation using RepeatMasker (v4.1.5) with the Fusarium repeat database (Dfam 3.7) identified a total of 8835 repeat elements spanning 545,741 bp, accounting for 1.17% of the total assembly. Simple repeats (7876 elements, 498,304 bp, 1.06%) and low complexity sequences (959 elements, 47,437 bp, 0.10%) constituted the entirety of the repetitive content. Notably, no transposable elements, including retroelements (SINEs, LINEs, LTR elements) or DNA transposons, were detected, suggesting a highly streamlined repeat landscape consistent with compact fungal genomes.

(A) Maximum likelihood phylogenetic tree based on ITS sequences, (B) Pairwise ANI heatmap based on whole-genome sequences (FungANI). Color scale from red (low) to green (high ANI); dashed line separates *F. proliferatum* from other *Fusarium* species.Genome size of *F. proliferatum* TQN5T (47.7 Mb) was comparable to other *F. proliferatum* strains (43.2 - 58.5 Mb), except for DSM106835 which exhibited a notably larger genome size. The total number of predicted genes in TQN5T (14,299) was within the range reported for other *F. proliferatum* genomes (14,054 - 16,509). Assembly contiguity varied considerably across strains, with N50 values ranging from 509,240 bp (TQN5T) to 4402,342 bp (Fp9), reflecting differences in sequencing technologies and assembly strategies employed (Table 2).

**Table 2:** Comparison of genome assembly statistics of *Fusarium proliferatum* TQN5T with publicly available *F. proliferatum* genomes.

No	Strain	Accession	Genome size (Mb)	GC%	Contigs	N50 (bp)	Genes	Host
1	*Fusarium proliferatum* TQN5T	GCA_033822135	47.7	46.26	443	509,240	14,299	*D. versipellis*
2	*Fusarium proliferatum* ET1	GCA_900,067,095	45.2	46	32	3311,891	16,509	Asparagus
3	*Fusarium proliferatum* NRRL62905	GCA_900,029,915	43.2	48.5	197	764,294	15,602	Maize
4	*Fusarium proliferatum* ITEM 2341	GCA_003290285	45.5	48.5	173	535,935	14,379	Date palm
5	*Fusarium proliferatum* Fp_A8	GCA_003615215	45.7	48.65	581	535,935	15,418	Onion
6	*Fusarium proliferatum* DSM106835	GCA_959,609,175.1	58.5	49	418	4383,091	15,580	Date palm
7	*Fusarium proliferatum* Fp9	GCA_018350245.1	43.9	48.28	12	4402,342	14,054	Rice

**Fig. 2:**
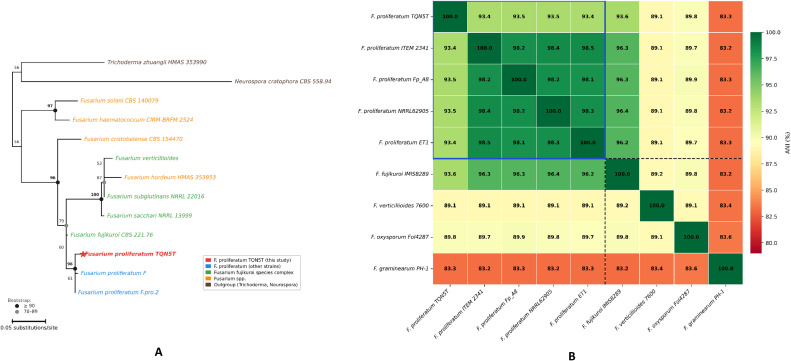
Phylogenetic analysis and average nucleotide identity (ANI) of *Fusarium proliferatum* TQN5T and related *Fusarium* species.

The maximum likelihood phylogenetic tree based on ITS sequences revealed that *F. proliferatum* TQN5T formed a well-supported clade with two other *F. proliferatum* strains (bootstrap = 96), confirming its taxonomic assignment to the species. *Fusarium fujikuroi* CBS 221.76 was placed as a sister group to the *F. proliferatum* cluster (bootstrap = 60), consistent with the close phylogenetic relationship between these two species within the *Fusarium fujikuroi* species complex. Members of the *F. fujikuroi* species complex, including *F. verticillioides, F. subglutinans*, and *F. sacchari*, formed a highly supported clade (bootstrap = 100), clearly separated from other *Fusarium* species such as *F. solani, F. cristobalense*, and *F. haematococcum. Trichoderma zhuangii* and *Neurospora cratophora* were placed as outgroups, providing a robust root for the phylogenetic analysis (Fig. 2A).

**Fig. 3:**
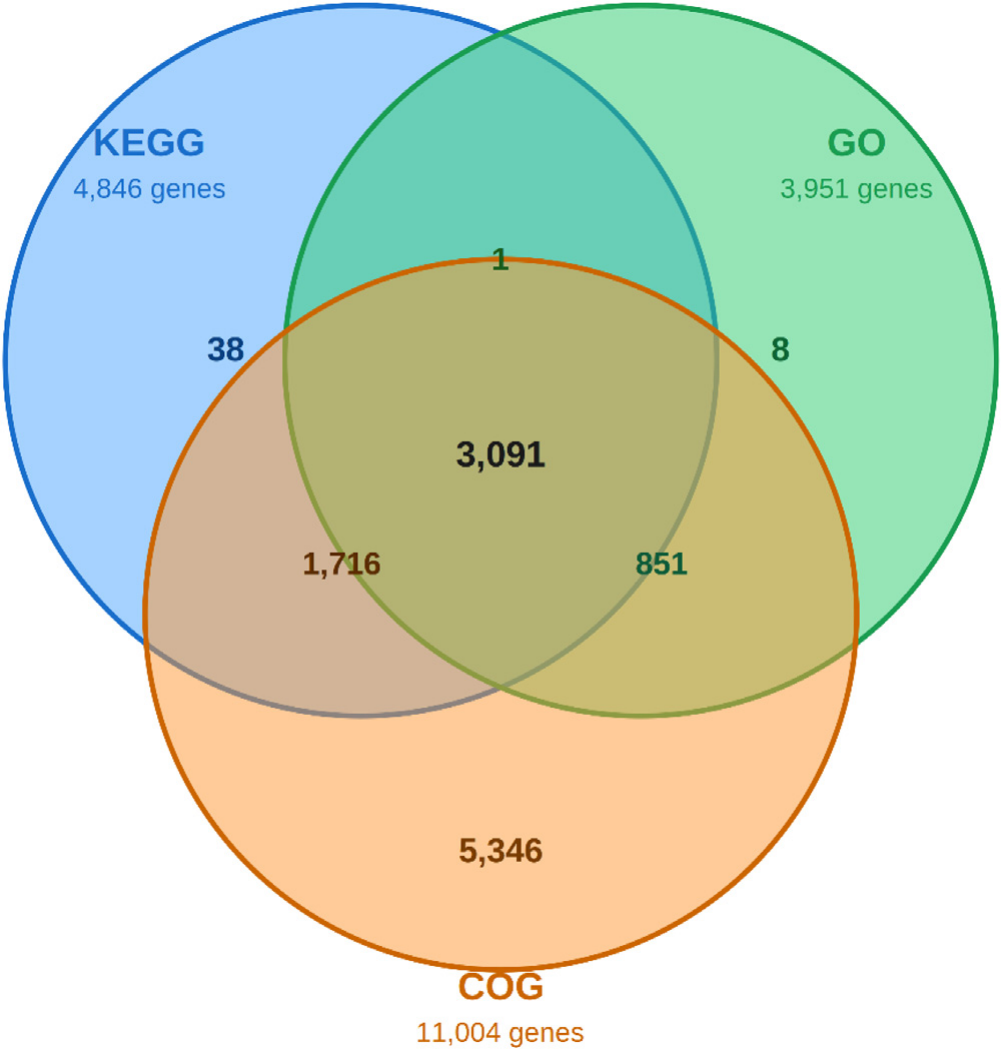
Venn diagram showing the number of shared and unique gene annotations across the KEGG, GO, and COG databases.

Whole-genome ANI analysis further supported the phylogenetic placement of TQN5T. Pairwise ANI values between TQN5T and other *F. proliferatum* strains were consistently around 93.4–93.5%. While ANI values showed limited resolution between *F. proliferatum* and the closely related *F. fujikuroi* species complex (93.6%), ITS sequence analysis revealed 99.43% similarity of TQN5T to *F. proliferatum* (MH793826.1), substantially supporting its species-level assignment. These ANI values, while slightly below the commonly accepted species boundary of 95 - 96% [[2], [3], [4]], remained substantially higher than those observed between TQN5T and more distantly related species, including *F. verticillioides* 7600 (∼89.1%), *F. oxysporum* Fol4287 (∼89.8%), and *F. graminearum* pH-1 (∼83.3%) (Fig. 2B). It is also noteworthy that comparable intraspecific ANI divergence has been reported in other *Fusarium* species; for instance, strains of *F. solani* exhibited ANI values of 93.7 - 94.8% while still being classified within the same species [5] and molecular sequence similarity values of 93.9 - 94.2% have been documented among closely related species within the *Fusarium concolor* complex [6]. The moderate ANI divergence of TQN5T may reflect its unique ecological niche as an endophyte isolated from *Dysosma versipellis* in Vietnam, as well as potential geographic isolation from other *F. proliferatum* strains predominantly reported from agricultural hosts in Europe and North America. Taken together, the convergent evidence from ITS-based phylogenetics (bootstrap = 96), ITS sequence similarity (99.43%), and whole-genome ANI analysis supports the robust classification of TQN5T as *Fusarium proliferatum* (Fig. 3).

Functional annotation of the 14,299 predicted coding sequences using eggNOG-mapper revealed that 11,051 genes (77.3%) were successfully assigned to at least one of three major functional databases. Among these, 11,004 genes (77.0%) were annotated with Clusters of Orthologous Groups (COG), 4846 genes (33.9%) were assigned to Kyoto Encyclopedia of Genes and Genomes (KEGG) pathways, and 3951 genes (27.6%) were classified with Gene Ontology (GO) terms. Venn diagram analysis revealed that 3091 genes (21.6%) were simultaneously annotated across all three databases, representing the core functionally characterized genes. A total of 1716 genes were shared between COG and KEGG, while 851 genes were shared between COG and GO, and only 1 gene was shared exclusively between KEGG and GO without COG annotation. The dominance of COG annotations over KEGG and GO reflects the broader taxonomic coverage of the COG database, which includes functional categories for uncharacterized and poorly characterized proteins. In contrast, the lower coverage of KEGG and GO annotations is consistent with the limited representation of Fusarium metabolic pathways and gene functions in these curated databases.

**Fig. 4:**
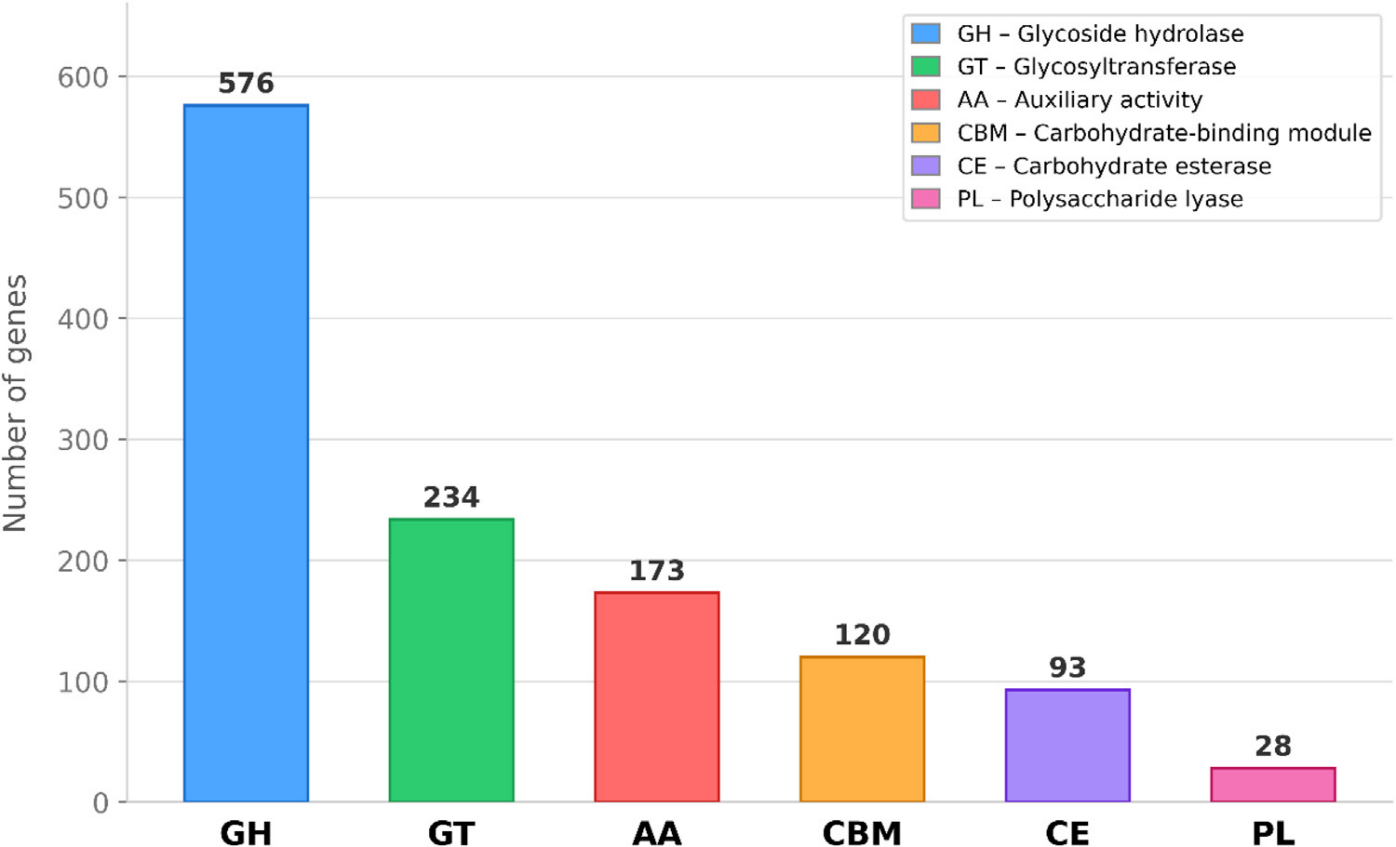
Distribution of carbohydrate-active enzyme (CAZyme) genes in *Fusarium proliferatum* TQN5T. GH, glycoside hydrolase; GT, glycosyltransferase; AA, auxiliary activity; CBM, carbohydrate-binding module; CE, carbohydrate esterase; PL, polysaccharide lyase.

Within the TQN5T genome, 60 regions were identified, harboring various types of smBGCs such as betalactone, cytokinin, fungal-RiPP-like, indole, isocyanide, isocyanide-nrp, NRP-metallophore, NRPS, NRPS-like, phosphonate, T1PKS, T3PKS, terpene, and terpene-precursor. The majority of detected BGCs showed no significant similarity to known clusters in the MIBiG database, highlighting the considerable potential for discovering novel secondary metabolites within the *F. proliferatum* TQN5T genome. T1PKS and terpene emerged as the most prevalent smBGC types. Among the 60 detected regions, 22 exhibited similarity to known clusters, with similarity confidence levels ranging from low to high. Clusters assigned high similarity confidence included koraiol, ACT-Toxin II, oxyjavanicin, fosfonochlorin, clavaric acid, α-acorenol, fusatin/fusatinic acid, choline, depudecin, gibepyrone-A, bikaverin, and mangicol A. α-Acorenol has been reported to possess antioxidant and anti-inflammatory activities (Fig. 4).

**Fig. 5:**
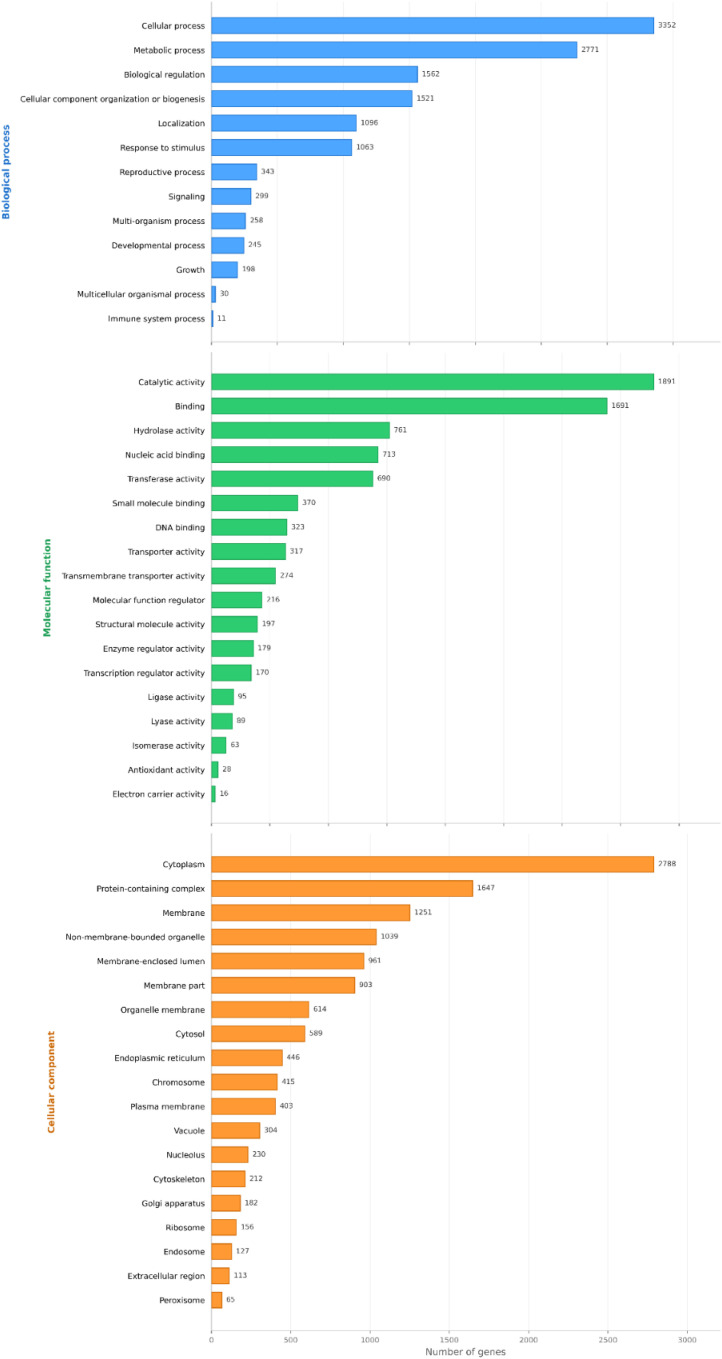
Gene Ontology (GO) annotation of predicted genes in *Fusarium proliferatum* TQN5T. GO terms were assigned at level 2 and categorized into three major functional categories: biological process (BP), molecular function (MF), and cellular component (CC).

The genome analysis of strain TQN5T revealed 1224 putative CAZy coding genes identified by the HMMER and DIAMOND algorithms and categorized into six classes. These classes consisted of 173 auxiliary activity (AA) genes, 93 carbohydrate esterase (CE) genes, 120 carbohydrate-binding module (CBM) genes, 576 glycoside hydrolase (GH) genes, 234 glycosyltransferase (GT) genes and 28 polysaccharide lyase (PL) genes (Fig. 5).

A total of 50 subcategories were delineated from GO terms, comprising 19, 18, and 13 subcategories for cellular component, molecular function, and biological process, respectively. Notably, cytoplasm (2788 genes) and cellular process (3352 genes) stood out as the most prevalent subcategories within cellular component and biological process, respectively, while catalytic activity (1891 genes) dominated the molecular function category. The comprehensive functional annotation of the entire genome sequence of *F. proliferatum* TQN5T unveils its extensive capacity for growth, transportation, metabolism, and environmental responsiveness, along with its ability to secrete compounds such as enzymes.

## 4 Experimental Design, Materials and Methods

### 4.1 Fungal strain isolation and culture

The endophytic fungal strain *Fusarium proliferatum* TQN5T was isolated from *Dysosma versipellis* collected in Ha Giang Province, Vietnam, and deposited at the Vietnam Academy of Science and Technology Culture Collection of Microorganisms (VCCM) under accession number VCCM 44,284. The strain was preserved at −20 °C for long-term storage and subcultured on potato dextrose agar (PDA) medium at 25 °C for experimental procedures. Isolation and cultivation were carried out according to the method previously described by [7].

### 4.2 DNA extraction and genome sequencing

The *F. proliferatum* TQN5T isolate from the root of *D. versipellis* was used for DNA extraction using the Quick-DNA Fungal/Bacterial MiniPrep™ Kit (Zymo Research Group, California, USA), following by the manufacturer's instructions. The DNA product was quantified using Pippin pulse—SAGE and QUBIT 3.0. The SMRTbell Express Template Prep Kit 2.0 (100–938–900, Pacific Biosciences, Menlo Park, CA, USA) and the Sequel Binding and Internal Ctrl Kit 3.0 (101–626–600) were utilized in sequencing library preparation. The subsequent fungal genome sequencing was conducted via PacBio SEQUEL system (Pacific Biosciences, Menlo Park, CA, USA) at the Institution of Biology, VAST.

### 4.3 Processing and genome assembly

Raw PacBio reads were processed using SMRT Link v10.2 (Pacific Biosciences). Quality control of raw subreads was performed using the default filtering parameters within the SMRT Link pipeline, including removal of short subreads (minimum subread length: 500 bp) and low-quality reads (minimum read quality score: 0.80). Adapter sequences were automatically trimmed during the subread extraction step. The filtered, high-quality subreads were subsequently used as input for genome assembly. Genome assembly was conducted using the Hierarchical Genome Assembly Process (HGAP4) [8]. Assembly quality was evaluated using two complementary approaches: BUSCO v5.8.0 with the fungi_odb10 database (1312 single-copy ortholog groups) was used to assess genome completeness [9], while QUAST v5.3.0 was employed to evaluate assembly statistics including contig number, total length, N50, and GC content [10]. Repeat sequences were identified and masked using RepeatMasker v4.1.5 with the *Fusarium* repeat database (Dfam 3.7) [11].

### 4.4 Genome annotation and functional analysis

Protein-coding genes were predicted using the BRAKER2 pipeline, utilizing GeneMark-ET and AUGUSTUS [12], and annotations were generated using TQN5T and *F. proliferatum* ET1 reference genomes (GenBank: GCA_900,067,095.1). tRNAs were predicted using tRNAscan-SE software version 2.0 [13], and rRNA was identified using barrnap software version 0.9 (https://github.com/tseemann/barrnap) [14]. Carbohydrate-active enzymes (CAZymes) were annotated using the CAZy database via dbcan2 meta server with diamond and hmmscan [15]. Secondary metabolite biosynthetic gene clusters (smBGCs) were annotated using antiSMASH version 8.0 (https://fungismash.secondarymetabolites.org/#!/start) [16]. Gene comparison and functional assignments for KEGG pathways, GO, and COG were performed using Eggnog-mapper [17].

### 4.5 Taxonomic classification

Taxonomic identification of TQN5T was performed by ITS-based rDNA sequencing, morphological characterization, and physiological analysis as previously described [18]. The ITS sequence of TQN5T (GenBank accession MZ496938.1) showed 99.43% similarity to *Fusarium proliferatum* (MH793826.1). For phylogenetic analysis, ITS sequences of 12 related fungal strains were retrieved from the NCBI GenBank database and aligned using MAFFT [19]. A maximum likelihood phylogenetic tree was constructed using IQ-TREE 2 with the best-fit substitution model selected by ModelFinder, and bootstrap support was assessed with 1000 replicates [20]. *Trichoderma zhuangii* (NR_202,778.1) and *Neurospora cratophora* (NR_159,859.1) were used as outgroups. Whole-genome Average Nucleotide Identity (ANI) was calculated using FungANI against publicly available *Fusarium* genomes to further confirm species-level assignment [3].

## Limitations

The ANI analysis showed limited resolution between *F. proliferatum* and *F. fujikuroi* due to their close genomic relatedness. Future multilocus phylogenetic analysis incorporating additional markers such as *TEF1-α* and *RPB2* would provide further support for species-level classification of TQN5T.

## Ethics Statement

The authors have read and follow the ethical requirements for publication in Data in Brief and confirm that the current work does not involve human subjects, animal experiments, or any data collected from social media platforms.

## CRediT Author Statement

**Thi Nhung Doan:** Methodology, Software, Formal analysis, Data Curation, Writing - Original Draft, Writing - Review & Editing; **Ha Thi Hong Nguyen**: Methodology, Sampling, Writing - Original Draft; **Quang Ho Tran**: Methodology, Investigation, Formal analysis, Data curation, Validation, Writing - Original Draft; **Tung Ngoc Quach:** Methodology, Data curation, Validation, Writing - Original Draft; **Ngoc Bich Pham**: Methodology, Investigation, Data Curation, Supervision, Writing - Review & Editing.

## Data Availability

NCBI Gene bankDraft genome of Fusarium proliferatum TQN5T isolated from Dysosma versipellis (Original data)
